# Political alignment, centralisation, and the sense of government unpreparedness during the COVID-19 pandemic^☆^

**DOI:** 10.1016/j.ejpoleco.2021.102144

**Published:** 2022-06

**Authors:** Massimiliano Ferraresi, Gianluca Gucciardi

**Affiliations:** European Commission, Joint Research Centre (JRC), Ispra, Italy

**Keywords:** Policy outcomes, Voter perception, Policy-making process, Centralisation, COVID-19

## Abstract

We rely on a periodic public opinion poll indicator of the performance of mayors collected for 103 large cities in Italy and in three waves (2015, 2017, and 2020) to examine whether and to what extent the exogenous shift in policy-making decisions induced by the COVID-19 pandemic has affected citizens' perceptions regarding attributions of responsibility. We leverage the variation in political alignment between central and local governments and implement a difference-in-differences research design, finding that when decisions are fully centralised (during the lockdown), voter approval for the mayor of an aligned city decreases by around 7%. Further analyses suggest that our results are more marked (i) during pre-electoral years and (ii) in cities with a lower level of social capital. Lastly, we document that the decrease in the approval ratings of aligned mayors is entirely guided by cities not severely hit by the pandemic, thereby reflecting a sense of ‘discontent’ in these areas for the policy decisions adopted by the central government to tackle the pandemic.

## Highlights

• We use data on the performance of the mayor, collected for 103 large cities in Italy.
• We exploit the change in policy decisions induced by the pandemic.
• We employ a DiD between cities politically aligned and non-aligned with the central government.
• When decisions are fully centralised the voter approval for the mayor of an aligned city decreases by of around 7%.
• These findings reflect a sense of ‘punishment’ for the lack of central government preparedness against the pandemic.

## 1 Introduction

Municipalities are at the heart of the Italian decentralised system of government. As in most other countries, they are responsible for important public programmes in the fields of welfare services, territorial development, local transport, infant schools, sports and cultural facilities, local police services, as well as infrastructure spending. These activities are easily recognised by citizens, who since 1993 have also been able to directly vote for the mayor and municipal council members. It then follows that citizens often assign a decisive policy role to the mayor as she represents the first point of reference for pursuing their interests or addressing their issues (Sancino and Castellani, 2016), thus enhancing citizens’ capacity to attribute policy responsibilities to the right level of government.

Nonetheless, on January 31^st^, 2020, the Italian central government proclaimed a national state of emergency, which entitled it to take any relevant measure to solve the crisis brought about by the COVID-19 pandemic. Since then, leveraging its increased centralised authority in order to control the reproduction rate of the new coronavirus, the government progressively announced several measures that closed schools and universities, public spaces, non-essential businesses and economic activities, along with restricting the movements of individuals (colloquially referred to as ‘lockdown’). All of these measures came into force identically over the whole territory.

While it is widely recognised in the literature that in a crisis a centralised decision-making system is more efficient than a multi-level political architecture of governance (Boin and ‘t Hart, 2003; ‘t Hart et al., 1993) because centralised decisions allow urgent responses to be implemented quickly (Bronner, 1982; Cohen, 1979; Perrow, 1967), the attribution of responsibility might become unclear and, therefore, voters cannot possibly understand the policy-making process in sufficient detail to accurately attribute responsibility for its outcomes (Anderson, 2006; Duch and Stevenson, 2008; Leyden and Borrelli, 1995; Lowry et al., 1998; Powell and Whitten, 1993; Royed et al., 2000; Treisman, 2007).

Evidently, there are complex mechanisms behind voter attributions of policy-making influence, but intuition would suggest that the clarity of responsibility is a key aspect—the point here being that voter perceptions of policy-making influence can be affected by the sharp change in the decision-making process, as occurred during the first wave of the COVID-19 outbreak.

It is this issue that we deal with here. In particular, we ask the following question: How do voters evaluate local politicians in response to a sharp change in the policy-making decision system that promotes a common policy response? Of course, one cannot hope to find an unambiguous answer to this central question, which has been the subject of a significant literature; one can, however, hope to find robust evidence and clarify some of the deeper forces at work regarding the causal link between policy-making decisions and voter attributions of policy-making influence by overcoming some empirical limitations detected in earlier studies, primarily related to lack of ‘causality’ (see Healy and Malhotra (2013) for a discussion of these limitations).

In this work, we exploit the change in the decision-making system induced by the pandemic to study voter attributions of policy-making influence. Specifically, we rely on a panel of 103 large cities in Italy (all provincial capitals) observed in three waves (2015, 2017, and 2020) to examine whether and to what extent the exogenous shift in the policy-making system affected citizens' perceptions of local politicians. To identify this effect, we take advantage of the political alignment of the city council with regard to the central government and implement a difference-in-differences research design. We posit that politically aligned cities are more influenced by the change in the decision-making process, for two alternative reasons. On the one hand, voters in these cities might find it more difficult to separate and clearly identify the attribution of activities and responsibilities between the central and local governments, seeing as they share the same ruling party, while for voters in non-aligned cities such responsibilities might be easier to separate (‘blind-spot’ hypothesis). On the other hand, citizens might perfectly identify policy responsibilities across levels of government and hence any effect detected at the local level simply reflects a positive or negative perception of the policies adopted by the central government during the pandemic (‘punishment or reward’ hypothesis).

To proxy the policy-making influence that each voter expects each mayor/municipal council to exert, we adopt the ‘Governance Poll’ indicator, a periodic public opinion poll on the approval ratings of mayors (and municipal councils). This indicator represents a local measure of ‘political’ performance as citizens evaluate mayors—and councils—not based on their perceptions of local conditions but according to actual local performance indicators. Therefore, we compare the difference in the Governance Poll score between aligned and non-aligned cities before the pandemic, when policy outcomes were unambiguously attributed to the local policy maker, with the same difference during the COVID-19 outbreak, when decisions were fully centralised.

Our results indicate that when decisions are in the hands of the local governments, the attribution of responsibility is not affected by their alignment status, thus revealing that citizens are perfectly able to punish or reward politicians at different levels of government for their actions (Fortunato et al., 2020). Conversely, the governance score achieved by an aligned city during the lockdown, when decisions were fully centralised, is about 7% lower compared to what it would have been in the absence of the lockdown, i.e. when policy decisions are in the local government's own hands. Our main results survive a number of robustness checks. Further analyses suggest that our findings are more marked (i) during pre-electoral years as compared to other years in a term and (ii) in cities with a lower level of social capital, whereas we do not find a more pronounced effect in cities guided by mayors supported by large majorities. Yet, we find suggestive evidence that the shrink in the Governance Poll indicator is associated with cities politically aligned only with the central government and not by those aligned with the regional government.

Finally, we document that the decline in the Governance Poll indicator is entirely driven by cities located in areas less affected by the pandemic. This evidence supports the hypothesis that voter perceptions of local government performance in aligned municipalities worsen not because citizens have ‘blind spots’ that cause them to misattribute policy responsibility; rather, such a worsening seems to be driven by a sort of ‘punishment’ directed towards the central government. This last finding resembles the ‘disillusion’ effect detected by Daniele et al. (2020) and might be interpreted as the perception by people in these areas, which were less plagued by the virus, of a lack of government preparedness against the pandemic. Since during crises citizens always overwhelm governing institutions to some degree, they may have had different expectations for the government's management and tackling of the pandemic.

Our work is mostly related to the strand of research focusing on the effects of alignment—here referring to incumbents of sub-national governments belonging to the same political party as the national one—on several policy outcomes. Along these lines, the first attempts to empirically investigate whether political alignment with the central government generates higher levels of discretionary grants to local governments are provided by Levitt and Snyder (1995) and Larcinese et al. (2006). More specifically, Levitt and Snyder (1995) rely on district-level data on election outcomes and federal assistance programs in US for the 1984–1990 period, finding that Democratic districts received more federal spending under the Carter administration than under the Reagan administration. In a similar vein, Larcinese et al. (2006) study the allocation of the US federal budget and show that states whose governors belong to the same party as the president receive more funds. A large literature developed from these works documenting and seeking to explain political alignment effects, not only considering grants or spending programmes (Bracco et al., 2019; Brollo and Nannicini, 2012; Gonschorek et al., 2018; Lara and Toro, 2019; Solé-Ollé and Sorribas-Navarro, 2008) but also bureaucratic performance (Gamalerio, 2020; Rivera, 2020) and public services (Callen et al., 2020). We complement this literature by exploiting political alignment as a way to assess whether citizens’ preferences regarding local politicians are affected by the change in the decision-making process resulting from the pandemic.

Furthermore, we contribute to the literature on retrospective voting, with a focus on approval ratings observed at regular intervals throughout a mayor's time in office (Fortunato et al., 2020) rather than electoral outcomes (see Healy and Malhotra (2013) for a comprehensive review). By leveraging variation stemming from a stochastic and unpredictable event, the COVID-19 outbreak, we also contribute to the literature that investigates government responses to natural disasters to identify the relationship between policy action and voter responses (e.g. Achen and Bartels, 2004; Bechtel and Hainmueller, 2011; Gasper and Reeves, 2011; Healy and Malhotra, 2013; Malhotra and Kuo, 2008; Masiero and Santarossa, 2021). Our focus also overlaps with the small yet growing strand of papers looking into the effects of the COVID-19 outbreak on political attitudes (Amat et al., 2020; Bruck et al., 2020; Bol et al., 2020; Bækgaard et al., 2020). We differ from most studies, which are primarily based on correlations, by providing more robust evidence grounded in a quasi-natural experimental approach. A notable exception is Daniele et al. (2020), who provide experimental evidence on a comprehensive set of socio-political attitudes by means of online surveys run at the country level. We therefore complement their analysis by relying on a more fine-grained indicator of voter perception at the local level. Our work is eventually tied to the literature documenting dissatisfaction with the political establishment during crises (Margalit, 2019), which includes studies from the US (Stevenson and Wolfers, 2011) and Europe (Hernandez and Kriesi, 2016). Lastly, our research also ties in with the emerging literature tackling the challenges of the COVID-19 outbreak in different areas of economics and political science (Brodeur et al., 2020, and the literature therein).

The remainder of this work is structured as follows. Section 2 illustrates the institutional context, Section 3 describes the data, and the econometric strategy while findings are presented in Section 4. Robustness tests are discussed in Sections 5. Heterogeneous effects are analysed in Section 6, while Section 7 further investigates the mechanism behind our findings. The last section offers some concluding remarks.

## 2 Institutional setting

### 2.1 Municipal decentralisation and municipal elections in Italy

The Italian Constitution defines four administrative layers of government: the central government, regions, provinces, and municipalities. While most regions and provinces are ruled by ordinary statutes, some (the autonomous regions and provinces) are ruled by special statutes.^1^ Furthermore, Italy counts 107 provinces, which were reformed by law 56/2014 reducing their public competences and eliminating the possibility of the direct election of their own representatives. Finally, municipalities are the smallest level of jurisdiction and number around 8000; the average size is around 6400 inhabitants, and most (approximately 90%) have fewer than 15,000 inhabitants.

Italian municipalities are responsible for a large array of important public programmes in the fields of welfare services, territorial development, local transport, infant schools, sports and cultural facilities, local police services, as well as infrastructure spending. As a share of the general government budget, in the timespan covered by our empirical analysis (2015–2020) municipalities accounted for about 8.5% of total public expenditure, on average, which corresponds to €66 billion per year. On the revenue side, municipalities can rely on transfers from higher levels of government (mainly the central and regional governments) and, as a result of a lengthy process of fiscal devolution, on their own revenue sources.

As for the municipal-level electoral system, since 1993 Italy has opted for a mayor–council system: the municipal council members and the mayor are separately and directly elected by citizens in elections normally held every 5 years. The mechanism of direct election implies that the mayor is endowed with strong powers over municipal politics (a basic feature of presidential government), even though the council retains the power to dismiss the mayor by means of a vote of no confidence (a basic feature of parliamentary government).

### 2.2 The COVID-19 outbreak and the role of institutions

On January 31st, 2020, through a resolution of the Council of Ministers,^2^ the Italian government declared a state of national emergency (*stato di emergenza nazionale*) as a result of the health risk associated with the first two Italian cases of COVID-19. This temporary emergency condition was originally introduced for six months (up to the end of July 2020). It was renewed for over two months (up to October 15th, 2020), was further extended to January 31st, 2021, and was finally prolonged to the end of 2021.^3^ The emergency condition provides the national government and the Department of Civil Protection with ‘extraordinary’ or ‘special’ powers. In particular, it allows the government to act in derogation on many aspects by the issuance of DPCMs (Decrees of the President of the Council of Ministers), legal acts that are directly emanated by the president of the Council of Ministers without the approval of parliament and which can only be issued in case of a state of emergency.^4^ Indeed, Article 120 of the Italian Constitution foresees that the government can exercise ‘substitute powers’ over local authorities such as regions, provinces, and municipalities in case of serious danger to public safety and security. Although the same article recognizes that the substitute powers should be exercised in compliance with the principles of subsidiarity and loyal collaboration between central and local authorities, the emergency measure establishes that local acts can be issued only subject to the issue of national acts. Consequently, once issued a DPCM prevails over any other local acts (Baldini, 2020). Hence, overall, the national government—and the president of the Council of Ministers, in particular—plays a central role in defining the policy options to combat the pandemic, while the local authorities see their executive powers temporarily reduced or even cancelled.^5^ In practice, during the first wave of the pandemic all powers mainly rested in the hands of the central government, which promoted a common policy response, i.e. a generalised lockdown, to tackle the spread of the virus.

## 3 Data

The empirical analysis is based on a dataset of Italian municipalities resulting from the combination of different archives publicly available from the Italian Ministry of the Interior, the Italian Ministry of the Economy, the Italian Statistical Office (ISTAT), the National Association of Italian Municipalities (ANCI), and *Il Sole 24 ore*, Italy's main economic newspaper. The dataset contains a full range of information for each municipality, organized into three sections: (1) data on the local public opinion poll; (2) electoral data, including the party affiliation of mayors elected during the period covered by the dataset and other personal characteristics of the mayors; (3) demographic and socio-economic data.

As previously mentioned, Italian municipalities differ along several dimensions and are also affected by many legislative thresholds based on population. To begin with, municipalities that are also provincial capitals normally provide a much wider range of services. Moreover, the salaries of the mayor, of the members of the executive committee, and of the councillors, the size of the city council and of the executive committee, the electoral rule, whether or not a municipality can have additional elective bodies in its districts, and whether or not a municipality can host hospital facilities or organize a healthcare district are all policy assignments that vary with population size (Gagliarducci and Nannicini, 2013). In addition, vertical transfers from the central government vary proportionally with population size (Law 504/1992). Furthermore, municipalities with fewer than 5000 inhabitants are exempted from having to comply with a set of rules imposed on municipalities by the national government in order to improve their fiscal discipline (based on the Domestic Stability Pact). Finally, the large majority of votes at the local level in Italy are cast in favour of independent lists (*liste civiche*), for which it is not possible to associate any political colour. As shown by Bracco et al. (2015), more than 65% of Italian municipalities cannot be classified as left or right. For this reason, they strongly recommend relying only on large municipalities to avoid biased estimates (ibidem, p. 83).

In light of these concerns and with the aim of clearly identifying effects, we restrict our sample to municipalities that are the capital of a province (*capoluoghi di provincia*). Such restrictions ensure that there are no other policy changes, structural reforms, or different institutional settings that are relevant for the municipalities in the sample, thus making possible unbiased comparisons. With these restrictions, we are left with almost the entire universe of cities that are capitals of a province, consisting of 103 municipalities and including 304 observations for the years 2015, 2017, and 2020.^6^

Looking at the political coalitions supporting the mayors, Table 1 documents that the absolute majority belongs to the centre–left wing (58%), while approximately one third (30%) can be attributed to the centre–right. As expected, the presence of independent lists is strongly reduced since we look at sufficiently large municipalities (provincial capitals). Indeed, only 7% of elected mayors are supported by *liste civiche*, with zero cases emerging in the case of regional capitals. The remaining councils (4%) are assigned to the Five Star Movement (*Movimento 5 Stelle,* M5S), a post-ideological party (Bordignon and Ceccarini, 2015) not directly attributable to more traditional political schemes.

**Table 1:** Political coalition supporting the mayor by municipality type.

Coalition	All municipalities		Regional capitals (*capoluoghi di regione*)		Provincial capitals (*capoluoghi di provincia*)	
	Number	%	Number	%	Number	%
Centre–left	178	58%	37	62%	141	57%
Centre–right	93	30%	16	27%	77	31%
M5S	11	4%	5	8%	6	2%
Independent (*lista civica*)	21	7%	0	0%	21	8%
Others	1	0%	0	0%	1	0%
Missing	5	2%	2	3%	3	1%
Total	309	100%	60	100%	249	100%

**Note**: The table reports statistics on mayors' coalition affiliations in the years of the poll (i.e. 2015, 2017, and 2020). The category ‘Others’ includes one centre coalition.

### 3.1 Dependent variable

Our dependent variable is the approval rating of the mayor expressed by the residents of the municipality. This information is retrieved from a periodic public opinion poll (called the ‘Governance Poll’) conducted by IPR Marketing, an Italian institute specialized in surveys, and published by the principal Italian daily financial newspaper, *Il Sole 24 Ore*. We collected this data for the last three years in which the survey was completed, i.e. 2015, 2017, and 2020. It is worth mentioning that in 2020, the Governance Poll was conducted in June, when the first wave of the pandemic had ended and, therefore, when citizens had already formed an opinion on how the COVID-19 outbreak was managed by the central and local governments.

The poll was conducted in all municipalities that are the capital of a province.^7^ Based on the IPR Marketing declaration, in each municipality the sample is composed of between 600 and 1000 citizens and is representative of the adult population with respect to gender, age, and area of residence. The interviews were conducted using a mixed system including (*i*) computer-assisted telephone interviewing (CATI) and (*ii*) computer-assisted web interviewing (CAWI).

The respondents answered the following question:

‘I would like to ask your overall opinion on the work of the mayor. If municipal elections were held tomorrow, would you vote in favour of or against the incumbent mayor?‘^8^

The indicator is then built as the percentage of respondents who expressed their potential intention to vote in favour of the incumbent mayor over the total number of respondents; therefore, it is a continuous variable ranging between 0 and 100. According to our sample, the average value of our dependent variable is approximately 53.6%, with a minimum score of 38.1%, a maximum of 69.9%, and a standard deviation of approximately 5%.^9^

The key aspect of this variable is that it captures citizen perceptions regarding the attribution of responsibility to the mayor and the municipal council. Stated differently, we can estimate the policy-making influence that each respondent expects each mayor/municipal council to exert under two different systems: one characterized by a high level of decentralisation (before the COVID-19 outbreak), where policy outcomes are unambiguously attributed to the local policy maker, and the other being substantially fully centralised (during the pandemic).

### 3.2 Aligned cities

Our treatment variable is given by the political alignment of the city council with the central government. For this purpose, we define the alignment variable, *Aligned*, as equal to 1 if the mayor's party/coalition is the same as that in power at the central level at the time the poll was conducted and zero otherwise.

**Fig. 1:**
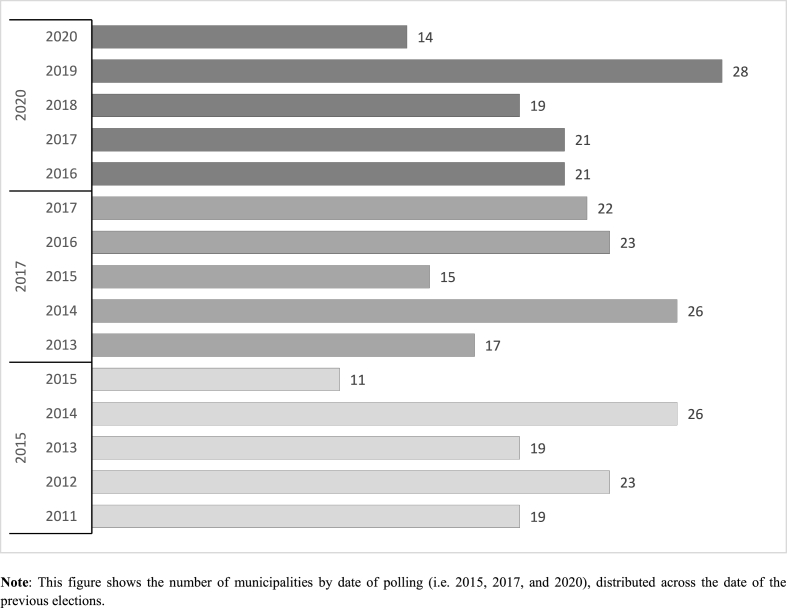
Number of elections by year of the poll and date of elections.

It is worth noting that there are two sources of variation in the city-alignment status. First, the city council might share the same political coalition as the central government because of local elections. Along these lines, and as previously mentioned, municipal elections are normally held every 5 years between April and June, but the timing is not the same for all municipalities. The staggering of electoral dates is the result of local governments having to resign before the end of their term because of not being able to form a majority in the city council supporting the local government or because of political scandals or judicial impeachment. It then follows that the staggered timing of the Italian municipal elections determines a sort of random assignment of the political cycles of municipalities, and therefore of the alignment status. That is to say, the position in the term of a single municipality—and its relative majority—in a given year can be considered as good as randomly assigned (Coviello and Gagliarducci, 2017; Ferraresi, 2020), especially with respect to the timing of the pandemic hitting Italy. Fig. 1 demonstrates that municipalities indeed follow different election schedules. Specifically, of the 103 municipalities surveyed in 2020, 21 (20%) voted four or three years before the poll (i.e. in 2016 and 2017), 19 (18%) two years before, 28 (27%) the year before, and the remaining 14 (14%) voted in the same year of the poll but after the date it was conducted. The same applies for 22 municipalities surveyed in 2017 and for 11 surveyed in 2015.^10^

On the other hand, a municipality might become aligned as a result of national elections. Along the timespan of our analysis, a general election was held in 2018, with an additional governmental reshuffle in 2019, thus leading to three different governmental coalitions. In particular, until the general elections of 2018 the majority of the national government was guided by a left-wing coalition. After the election and up to September 4th, 2019, the national government was guided by the Five Star Movement (*Movimento 5 Stelle*) and the Northern League (*Lega Nord*), a far-right party. Following the governmental reshuffle, in September 2019 the majority was taken by a left-wing coalition composed of the Five Star Movement, the Democratic Party (*Partito Democratico*), and some other minor centre–left parties.^11^

Table 2 presents information on the number of elections by year and by winning coalition for aligned and non-aligned governments. It is interesting to note that the sample is not equally split between aligned and non-aligned municipalities, as the former group of municipalities (182 observations) is slightly larger than the latter (122 observations).

**Table 2:** Distribution of elections by aligned and non-aligned municipalities.

Year of the poll	Aligned					Not Aligned					Total poll
	Centre-right	Centre-left	M5S	Others	Total	Centre-right	Centre-left	M5S	Others	Total	
2015	0	73	0	0	73	19	0	3	3	25	98
2017	0	67	0	0	67	22	0	4	10	36	103
2020	0	38	4	0	42	52	0	0	9	61	103
Total	0	178	4	0	182	93	0	7	22	122	304

**Note**: The category ‘Others’ includes 21 independent lists (‘*liste civiche’*) and one centre coalition. Data on polls are not available for five municipalities in 2015.

### 3.3 Control variables

The dataset also includes some time-varying control variables that account for differences among municipalities in terms of their population structure and economic conditions. The demographic and socio-economic controls include total population (*population*), the share of population aged between 0 and 5 (*child*), the share of population over 65 (*aged*), and the share of foreign population (*foreigners*) as these variables can capture the presence of scale economies in the provision of public goods and also account for some specific age-related public needs such as nursery schools and nursing homes. Moreover, to proxy the average level of municipal education we adopt the share of peopled enrolled at university (*education*). Since the local economic conditions might influence perceptions of mayoral activities, we also factor in the unemployment rate (*unemployment rate*) and the number of local productive units for every 100,000 inhabitants (*firms*).^12^ Finally, the personal characteristics of the mayors may affect their approval ratings, while also being correlated with the alignment status. Therefore, in further specifications we account for the mayor's age (*age*), gender (*gender*), education (*edu*), past occupation (*profession*), and vote margin in the last election (*vote margin*), measured in vote shares.^13^

Before moving to the empirical investigation, as a preliminary piece of evidence it is worth noting that before the pandemic the average value of the municipal Governance Poll score obtained by aligned cities (54.029) was higher than that of non-aligned ones (52.869), with a difference equal to 1.160. Since the COVID-19 outbreak, when policy decisions became centralised, the same difference has become negative (−1.914). It then follows that the difference in the differences (−3.074 = −1.914 – 1.160) is statistically significant at the 5% level, suggesting that during emergency times the approval of local government actions drastically reduces when central and local governments are nested, that is, when they belong to the same party coalition.

## 4 Empirical strategy and findings

Since we are interested in understanding how and to what extent voters’ attributions of policy-making influence can be affected when decisions mainly rest in the hands of the central government, we consider politically aligned cities as treated and non-aligned cities as controls. We then exploit the change in policy decisions induced by the pandemic that led to a centralised decision-making system. The exogenous change allows us to compare the difference in the Governance Poll score between aligned and non-aligned cities before the pandemic, when municipalities could enjoy the usual discretion in setting policy decisions, with the same difference after the COVID-19 outbreak, when decisions were centralised.

The difference in differences (DiD) model estimated in this study is specified as follows:

(1) $$Governancepollscore_{it}=\alpha +\beta Aligned_{it}+\lambda Post_{t}+\gamma Aligned_{it}\times Post_{t}+f_{t}+f_{i}+u_{it},$$

where $Governancepollscore_{it}$ is the (log) of the Governance Poll score for city *i* at time *t*, where t=2015,2017,2020. *Aligned*_*it*_ is a dummy variable that takes a value of one if the mayor's coalition coincides with the coalition in power at the central level and zero otherwise; *Post*_*t*_ is a binary variable that is equal to one for the poll conducted in 2020, as a consequence of the COVID-19 pandemic; $f_{i}$ is an unobserved municipality-specific effect; $f_{t}$ are poll-year fixed effects that capture shocks common to every city in the year the survey was conducted; $u_{it}$ is the error term, clustered at the city level.^14^

It is important to note at the outset that in this estimation framework the coefficient *β* accounts for the impact of being aligned on the Governance Poll score before the COVID-19 outbreak as compared to not being politically aligned, while *γ* captures the differential effect with respect to *β* of being aligned during the outbreak. It then follows that the estimate of the combination of β+γ accounts for the difference in the Governance Poll score between aligned and non-aligned cities during the lockdown.

### 4.1 Findings

The first round of results is shown in Table 3. Each of the four columns correspond to a different specification of Eq. (1). The baseline specification, which includes municipality and year fixed effects, is reported in column (1). The model in column (2) factors in all demographic and socio-economic covariates described in Section 3.3, to control for characteristics of the municipalities that vary across time and space and are potentially correlated to the alignment status and the governance indicator. Column (3) allows for the personal characteristics of the mayors to be accounted for. Since Italian regions are involved in the decision-making process of some relevant public functions, i.e. health, education, and assistance, one might argue that there could be some other unobservable characteristics related to the specific region that might influence people's perceptions across time, thus affecting the municipal governance score. Along these lines, column (4) includes a set of region-by-year fixed effects to account for unobservable region-specific characteristics that vary over time.

**Table 3:** Baseline results.

Dep. variable	(Log of) Governance Poll score			
	(1)	(2)	(3)	(4)
Aligned × Post	−0.068** (0.029)	−0.064** (0.029)	−0.054** (0.028)	−0.066* (0.037)
Aligned	0.007 (0.024)	0.009 (0.025)	−0.020 (0.024)	−0.013 (0.028)
Post	0.018 (0.020)	0.030 (0.049)	0.006 (0.049)	0.033 (0.079)
				
Observations	304	304	304	304
Adjusted R-squared	0.033	0.066	0.242	0.253
Municipality FE	Yes	Yes	Yes	Yes
Poll FE	Yes	Yes	Yes	Yes
Eco-socio-demographic controls	No	Yes	Yes	Yes
Political controls	No	No	Yes	Yes
Region × Poll	No	No	No	Yes

**Note**: *Aligned* is a dummy variable that takes a value of one if the political party of the mayor belongs to the same political sphere as the national government and zero otherwise. *Post* is a dummy variable that takes the value of one for the 2020 poll (during the pandemic) and zero otherwise. *Aligned x Post* is an interaction term equal to one for each municipality governed by a mayor politically aligned with the national government during the pandemic and zero otherwise. *Eco-socio-demographic* control variables are population, children, aged, population density, foreigners, share of population enrolled at university, unemployment rate, and number of firms. *Political* control variables are the mayor's age, gender, education level, profession, and distance in terms of vote share to the first non-elected candidate. Standard errors clustered at the municipality level are shown in parentheses. ***, **, and * indicate significance at the 1%, 5%, and 10% level, respectively.

The results are shown in Table 3. Central to the issue at hand is the coefficient of $Aligned_{it}\times Post_{t}$, which captures the differential effect of being politically aligned vs not being aligned during the pandemic, minus the same difference before the pandemic. The coefficient turns out to be negative, remarkably similar in magnitude (ranging from −0.054 to −0.068), and statistically robust across all specifications. It is also interesting to point out that a comparison of the estimates in columns (1) through (3) indicates that both groups of controls (demographic-socio-economic and political) play an important role, as γ in column (1)—where no controls are considered—is equal to −0.068, while it drops to −0.054 when all controls are included. In terms of point estimates, in column (1) it emerges that the governance score of an aligned city when decisions are fully centralised is about 7% lower compared to the score it would have obtained in the absence of a lockdown, when it is itself responsible for policy decisions.

What all of this seems to point to is that under a centralised decision-making system citizens of aligned municipalities seem to be less able to identify who to blame or praise for policy outcomes. There is, of course, an alternative explanation for this result, one that relies on the fact that citizens perfectly identify policy responsibilities across levels of government, but in aligned municipalities the observed decrease in the governance score might simply reflect negative perceptions of the policies adopted by the central government during the pandemic. This is what we explore further in Section 7.

## 5 Robustness tests

In this section, the validity of the previous results is confirmed by a battery of robustness tests that are intended to address possible issues related to the research design that could bias the baseline estimates. All of these results are reported in Section B of the Online Appendix.

To begin with, as outlined in Section 3.2 our framework allows three types of municipalities to be included: (i) always aligned; (ii) never aligned; and (iii) switchers. While a key trait of our identification strategy exploits the municipal variation in the aligned status due to the result of electoral competition (both at the national and at the local level), one might argue that in practice we do not observe the same city (aligned or non-aligned) before and during the COVID-19 outbreak, as one would in the standard DiD approach. In turn, this might create a potential bias in the estimates as municipalities in the treatment and control groups differ from one year to another. To mitigate such a concern, we create a subsample of municipalities composed of (i) cities that never change their status of aligned (*always aligned*) and (ii) municipalities that have never been aligned with the central government over the period of observation (*never aligned*). While this restriction limits the sample size, it makes it possible to compare our outcome variable in the same group of aligned municipalities before and after the pandemic with the difference in the same group of non-aligned ones, thereby eliminating any source of biased comparison. Results are reported in Table B1 and all of the baseline findings are fully confirmed, with the γ coefficient being slightly larger in magnitude (around 9%).

Second, a common way to conduct a placebo test in the context of DiD analysis is to focus on the span prior to the shock—that is, to simulate what would have happened to the Governance Poll score in aligned municipalities if a fake year were used for the pandemic. In our framework, we replicate the baseline model by supposing that the COVID-19 outbreak had occurred in 2017. In other words, we create a (*Fake*)*Post* dummy variable equal to one for the polls conducted in 2017 and zero otherwise, and we interact it with the aligned indicator. Were the coefficient associated with $Aligned_{it}\times \left(Fake\right)Post_{t}$ negative and significant, it would suggest that before the true year of the *de facto* centralised decision-making system experienced by Italian municipalities during the COVID-19 outbreak, aligned municipalities were already experiencing a lower governance score as compared to non-aligned ones, thus casting doubt on the validity of the previous results. Reassuringly, the placebo exercise does not lead to any statistically significant effect on our main outcome variable, as the *γ* coefficient turns out to be indistinguishable from zero in all specifications of the full sample (Panel A, Table B2) and also in the case where the subsample of always vs never aligned municipalities is used (Panel B, Table B2).

As was already alluded to, our dataset covers three years of the survey, two of them falling before the COVID outbreak (2015 and 2017) and another in the year of the pandemic (2020). Hence, as a further placebo check one could test whether there is a differential effect of being politically aligned in 2015 and in 2020 as compared to 2017 (baseline year). In practice, we estimate a slightly modified version of Eq. (1):

(2) $$Governancepollscore_{it}=\alpha +\delta Aligned_{it}+\beta Aligned_{it}\times Year_{2015}+\lambda Year_{2015}+\gamma Aligned_{it}\times Year_{2020}+\pi Year_{2020}+f_{i}+u_{it},$$

where β captures the differential effect on the Governance Poll indicator of being a city politically aligned with the central government in 2015 as compared to 2017 and γ accounts for the same difference of being politically aligned with the central government in 2020 with respect to 2017. Were our design well specified, we should not observe any significant effect associated with β, while γ should be negative and statistically significant. Shown in Table B3 of the Online Appendix, the coefficient of $Aligned_{it}\times Year_{2015}$ turns out to be indistinguishable from zero in all specifications, both in the full sample (Panel A) and in the restricted sample of always vs never aligned municipalities (Panel B). Conversely, the coefficient associated with $Aligned_{it}\times Year_{2020}$ is negative and statically significant in the two sub-samples (Panels A and B), thus confirming the validity of the research design. The only exception is in column (4), where we control for both economic, social and economic characteristics of the city and for and mayors' individual traits, as well as including a specific trend for each region. In this case, while the coefficient is negative, it is not statistically significant (p-value = 0.145). It is very likely, indeed, that the inclusion of several control variables and fixed effects reduces the efficiency of the estimator in small sample. Similar conclusions hold for column (8), when the analysis is replicated on the sub-sample of always vs never-aligned municipalities.

Another element that needs careful consideration is the true exogeneity of the alignment status. Notwithstanding that the pandemic could not have influenced the alignment status as local elections for some cities originally scheduled in May/June 2020 were postponed to September/October 2020, a remaining concern is that the treatment is not randomly allocated across cities. In other words, the ‘alignment’ status could be correlated with other municipality characteristics that in turn are influenced by the pandemic. If this were the case, the exogeneity conditions of the included control variables would be violated after the COVID-19 outbreak, leading to biased estimates. To overcome this concern, besides the inclusion of time-varying municipality and mayor characteristics we allow for their interaction with the *post* event indicator. This helps rule out that results are driven by changes in observable municipality characteristics and not by the pandemic event itself. These results are reported in Table B4 of the Online Appendix. In column (1), where we use the (log of) the Governance Poll score as the dependent variable, it turns out that the coefficient associated with $Aligned_{it}\times Post_{t}$ remains negative (−0.043) and statistically significant at the 10% level. In column (2), we restrict the sample to municipalities always aligned against those never aligned and, in this case, while it is negative (−0.043), the coefficient is no longer statically significant (p-value = 0.276). However, it is worth noting that the inclusion of several fixed effects, as well as many control variables (and their interaction with *Post*), could undermine the efficiency of the estimator in small samples. In our case, we indeed control for municipality and year fixed effects, together with the *Aligned* and *Post* variables—including their interaction—and we include 12 time-varying covariates and their interaction with *Post*. Thus, it is very likely that the not statistically significant effect is driven by the limited size of the subsample of always vs never aligned municipalities (134 observations). The same conclusions hold when we use the Governance Poll score indicators in percentage terms (see columns (3) and (4) of Table B4).

Furthermore, while in the baseline estimates we control for time-invariant unobserved determinants of the governance score by including municipality fixed effects, there still might potentially be some remaining sources of bias due to unobserved confounders. The usual way to overcome this issue is to add variables as controls on the right-hand side of the regression. In this case, if the presence of unobserved effects were relevant the coefficient of interest would be sensitive to the inclusion of these controls. However, these demographic, socio-economic, and political variables might be poorly measured proxies of the confounders. As recently shown by Pei et al. (2019), a more sensitive test consists of including individual controls as dependent variables on the left-hand side of the regression equation. Table B5 shows that of these balancing regressions for various demographic, socio-economic, and political controls, none yield significant effects. These results help rule out the possibility that any correlation between the *Aligned* variable and other time-varying characteristics of cities is driving the results.

As a final robustness test, we check whether our main findings are sensitive to the exclusion of a single city, given their relatively low number in our sample. For this reason, we have estimated Eq. (1) dropping one city at a time. The result of the estimated coefficient, γ, and its 95% confidence interval are shown in Figure B1, and the results are very similar to those obtained in our baseline specification. Hence, it can be concluded that our main results are not driven by a particular city and are thus generalizable.

To sum up, the analyses carried out in this section have strengthened the evidence of a negative relationship between municipality alignment and governance score during the pandemic, namely, when the policy decision process is entirely in the hands of the central government. In addition, the results indicate that it is very likely that such an effect is due to the shock caused by the COVID-19 outbreak, as no other plausible explanations that clearly hold as arguments against a causal interpretation of this relationship are found.

## 6 Heterogeneous effects

To investigate whether there is evidence of a heterogeneous response across cities, we analyse how the effect varies along several dimensions. It turns out our findings are more marked (i) during pre-electoral years as compared to other years of the term and (ii) in cities with a lower level of social capital, while we do not find a more pronounced effect in cities guided by mayors supported by large majorities (more visible). Moreover, we find support—at least by means of suggestive evidence—that the decline in the Governance Poll indicator is driven by cities politically aligned only with the central government and not also with the regional one. All these results are described and reported in Section C of the Online Appendix.

## 7 Citizen blind spots or disapproval of the central government

So far, we have shown that once the decision-making process is centralised the Governance Poll indicator declines more in aligned municipalities compared to non-aligned ones. Nevertheless, it is not yet clear whether such a decrease is because citizens are temporarily unable to accurately attribute responsibility for policy outcomes given the new institutional framework experienced during the pandemic, or instead, if the drop in the governance indicator reflects a negative perception of the policies adopted by the central government during the COVID-19 outbreak. Along these lines, when consequences of government's actions or inactions are perceived to be deleterious voters tend to be ‘disaffected’, especially during crises (Gregory, 2003, pp. 557–558).

To test whether the negative effect is due to discontent with the lack of central government preparedness, we exploit the intensity of the first wave of the pandemic across cities. The intuition is that citizens might have had different expectations regarding the policies taken by the government to tackle the pandemic depending on the severity with which the virus hit the area where they live. Therefore, if the observed decline in the Governance Poll indicator is due to a negative perception of the Government, one would expect a more marked decrease in relation to the intensity of the COVID-19 outbreak in cities that are politically aligned with the Government.

To measure the intensity of the pandemic, we follow Borri et al. (2021) and we rely on three indicators: (i) the excess mortality, (ii) the number of COVID-19-related cases (over the population), and (iii) lockdown intensity.^15^ For each of these, we use the median value to divide municipalities into those with high and low exposure to the pandemic (*HCovidExposure*)—namely those with a value above the median and those below—and then we interact this indicator with our *Aligned* and *Post* variables in a triple difference (DDD) model that takes the following form:

(3) $$Governancepollscore_{it}=\alpha +\beta Aligned_{it}+\lambda Post_{t}+\gamma Aligned_{it}\times Post_{t}+\rho Post_{it}\times HCovidExposure_{i2020}+\theta Aligned_{it}\times HCovidExposure_{i2020}+\delta Aligned_{it}\times Post_{t}\times HCovidExposure_{i2020}+f_{t}+f_{i}+u_{it},$$

where $Aligned_{it}\times Post_{t}$ accounts for the differential impact of being politically aligned with the central government during the pandemic as compared to cities not politically aligned, while $Aligned_{it}\times Post_{t}\times HCovidExposure_{i2020}$ captures the differential effect of being politically aligned for cities that have been severely hit by COVID-19 with respect to less exposed cities during the first wave of the pandemic. In practice, the impact of the centralisation of policies (i.e. the generalised lockdown) on the Governance Poll indicator for cities politically aligned with the central government that were less affected by the pandemic is given by $Aligned_{it}+Aligned_{it}\times Post_{t}$, while the same effect for cities highly affected by the spread of the virus is given by $Aligned_{it}+Aligned_{it}\times Post_{t}+Aligned_{it}\times Post_{t}\times HCovidExposure_{i2020}$.

Central to the issue at hand is therefore δ, which might provide some insights into the mechanism behind our results. In particular, there are three possible cases.

• δ = 0: no differential effect in politically aligned cities in relation to the degree of exposure to the pandemic. Were this the case, it would be likely that the centralisation of policy-making decisions triggered by the pandemic brought about citizen confusion regarding policy responsibilities between different levels of government.
• δ > 0 (and γ < 0): *Too stringent responses.* The decline in the Governance Poll indicator for aligned cities in less exposed areas is more marked than that observed for politically aligned cities in areas severely hit by COVID-19. In this case, such a decrease might reflect a sort of ‘punishment’ for the policy decisions of the central government. It is very likely that citizens in these areas found the policy responses of the Government (the generalised lockdown) to be too stringent and hence not really adequate for the actual pandemic situation experienced in the areas where they live.
• δ < 0 (and γ < 0): *Too soft responses.* The decline in the Governance Poll indicator for aligned cities is more pronounced in highly exposed areas than in less-affected ones. Citizens in areas severely affected by the COVID-19 outbreak might have had higher expectations regarding the policy responses of the government to the pandemic. In other words, more severe measures such as the further closure of other economic activities besides those that were forced to close during the first lockdown might have been preferred by citizens living in these areas.

Turning to the results, it emerges that the ‘punishment’ channel for a *too stringent response* is likely to be the most plausible explanation for the decline in the Governance Poll indicator. Indeed, in column (1) of Table 4—where we proxy the intensity of the pandemic with excess mortality—it turns out that the Governance Poll indicator significantly declines for aligned cities but only in areas not severely affected by the COVID-19 outbreak, as the coefficient of $Aligned_{it}\times Post_{t}$ is negative (−0.120) and statistically significant at the 1% level. Conversely, the coefficient associated with $Aligned_{it}\times Post_{t}\times HighMortality_{i2020}$ is positive (0.128) and statistically significant at the 5% level, thereby offsetting the negative coefficient found for the group of cities not strongly affected by the pandemic.

**Table 4:** Governance Poll score, alignment, and the severity of the pandemic.

Dep. variable	(Log of) Governance Poll score		
	(1)	(2)	(3)
Aligned × Post	−0.120*** (0.041)	−0.110** (0.044)	−0.087** (0.034)
Aligned	−0.001 (0.032)	0.001 (0.034)	−0.012 (0.034)
Post	0.029 (0.050)	0.015 (0.055)	0.023 (0.053)
Aligned × Post × High mortality	0.128** (0.052)		
Aligned × High mortality	−0.050 (0.046)		
Post × High mortality	−0.069* (0.036)		
Aligned × Post × High COVID-19 cases		0.112** (0.053)	
Aligned × High cases		−0.048 (0.044)	
Post × High cases		−0.042 (0.037)	
Aligned × Post × High restrictions			0.094* (0.050)
Aligned × High restrictions			−0.021 (0.047)
Post × High restrictions			−0.033 (0.036)
			
Observations	304	304	304
Adjusted R-squared	0.258	0.254	0.250
Municipality FE	Yes	Yes	Yes
Poll FE	Yes	Yes	Yes
Eco-socio-demographic controls	Yes	Yes	Yes
Political controls	Yes	Yes	Yes
Region × Poll	No	No	No

**Note**: *Aligned* is a dummy variable that takes a value of one if the political party of the mayor belongs to the same political sphere as the national government and zero otherwise. *Post* is a dummy variable that takes a value of one for the 2020 poll (during the pandemic) and zero otherwise. *Aligned x Post* is an interaction term equal to one for each municipality governed by a mayor politically aligned with the national government during the pandemic and zero otherwise. *High mortality* is a dummy variable that takes a value of one for those municipalities whose excess mortality rate observed in the first half of 2020, as compared to the (average in the first half of) 2015–2019, is higher than the median and zero otherwise. *High cases* is a dummy variable that takes a value of one for those municipalities in which the number of COVID-19 cases in the first half of 2020 is higher than the median and zero otherwise. *High restrictions* is a dummy variable that takes a value of one for those municipalities in which the share of inactive workers due to the government's COVID-related ‘lockdown’ restrictions is higher than the median and zero otherwise. *Eco-socio-demographic* control variables are population, children, aged, population density, foreigners, share of population enrolled at university, unemployment rate, and number of firms. *Political* control variables are the mayor's age, gender, education level, profession, and distance in terms of vote share to the first non-elected candidate. Standard errors clustered at the municipality level are shown in parentheses. ***, **, and * indicate significance at the 1%, 5%, and 10% level, respectively.

In practice, during the first wave of the pandemic voters of cities politically aligned with the central government responded asymmetrically to the measures taken by the government in relation to the severity of their experience of the pandemic. While the Governance Poll indicator reduces by approximately 12% in areas not strongly hit by COVID-19, the same coefficient turns out to be indistinguishable from zero for cities highly affected by the pandemic.^16^ Similar conclusions hold if we use the number of COVID-19 cases (column (2)) and the share of non-active population during the lockdown (column (3)).

What emerges, therefore, is that in a pandemic, when the policy-decision process is mainly exercised by the central government and the same policy response is applied to all areas of the country, citizens' approval ratings of mayors in aligned municipalities is significantly lower compared to that in non-aligned ones. Strikingly, this is not because citizens suffer from ‘blind spots’ that cause them to misattribute policy responsibility; rather, such a decrease seems to be driven by a sort of ‘punishment’ for the policy decisions of the government, which might reflect a sense of a lack of government preparedness against the pandemic, especially in light of the different experiences that citizens have regarding the spread of the virus. Along these lines, the asymmetric voter responses to the intensity of the pandemic seem to suggest that citizens in less-affected cities might have found the generalised lockdown to be a too stringent policy solution, thereby indicating that more targeted and differentiated measures to contrast the COVID-19 outbreak could likely have been implemented in the eyes of the citizens. Conversely, in cities highly affected by the spread of the virus, voter expectations regarding the policies adopted by the government appear to be met.

## 8 Conclusions

In this paper, we investigated how and to what extent voter attributions of policy-making influence are affected by the structure of the government. To induce a source of plausible exogenous variation, we exploited the fact that during the first wave of the COVID-19 pandemic the government decision-making process became highly centralised. The unprecedented nature of this event has allowed us to compare the governance score, capturing the level of citizen satisfaction with the work of the mayor and of the municipal council in politically aligned and non-aligned cities before the pandemic, when the mayor and the city council could enjoy the usual discretion in setting policy decisions, with the same difference during the COVID-19 outbreak when policy decisions were centralised.

Our findings indicate that the governance score of an aligned municipality when decisions are centralised is 7% lower compared to what it would have been in the absence of a lockdown, when policy decisions are in its own hands. All results survive a battery of robustness tests. Further investigations suggest that our findings are more marked (i) during pre-electoral years as compared to other years of a term and (ii) in cities with lower levels of social capital, while we do not find more a pronounced effect in cities guided by mayors supported by large majorities. Moreover, we document that voter perception of local governance in aligned municipalities decreased not because citizens misattributed policy responsibility to different government layers but rather because they were ‘punishing’ policy decisions underpinned by the central government.

Although our results are based on a robust indicator of citizen perceptions, it is possible that voter responses could represent a better and more objective indicator of voter attributions of responsibility. Along these lines, a natural extension of this analysis is to explore whether similar effects are observed when using electoral outcomes. In this regard, we conducted a preliminary exploration of this question by collecting data on the results of electoral competitions in the 16 cities that held elections in September/October 2020, i.e. during the pandemic. Among these 16 cities, 8 were led by a mayor politically aligned with the central government. It is interesting to observe that two cities experienced a swing from a left-to a right-wing coalition and were therefore no longer aligned with the central government. In three cases, cities continued to be aligned with the central government, but the share of votes cast for the ruling coalition decreased by approximately 3 percentage points as compared to the previous election, while in the remaining three cases the share of votes for the ruling (aligned) party was unchanged. When more electoral results become available for a larger sample of cities, future work could therefore apply our empirical strategy and investigate whether and to what extent voting decisions are affected by the sharp changes in the decision process induced by the COVID-19 outbreak.

Despite this limitation, these findings suggest that politically aligned cities appear to be more affected by citizen perceptions of the government's response to a crisis. It is very likely that citizens might have had different expectations regarding the policy responses of the Government to the pandemic, and the decline in the Governance Poll indicator in cities politically aligned with the government reveals that such expectations, at least for a portion of voters, were not met. In other terms, if policies adopted by the central government are perceived as unpopular—as was the case in those areas less affected by the spread of the virus—or if there is an impression of a lack of government preparedness against the pandemic, such a (negative) perception is exacerbated in cities whose mayor has the same political affiliation as the central government.

## Declaration of competing interest

The authors declare that they have no relevant or material financial interests that relate to the research described in this paper.

## Data Availability

Data will be made available on request.

## Footnotes

1 Italy has five autonomous regions (Sicily and Sardinia, which are insular territories, and Valle d’Aosta, Trentino-Alto Adige, and Friuli-Venezia Giulia, which are northern boundary territories) and two autonomous provinces (Trento and Bolzano).

2 See https://www.governo.it/it/coronavirus-misure-del-governo.

3 We should acknowledge that by law (Codice della Protezione Civile—Legislative Decree no. 1, January 2nd, 2018), a state of national emergency may be declared for a maximum of 12 months and can be extended up to another 12 months. Therefore, in principle, it may be further prorogated up to January 31st, 2022, under critical circumstances.

4 Given the prolonged extraordinary circumstances, after a first preliminary phase the president of the council was asked to present the contents of DPCMs to the Italian Parliament before their approval.

5 Along these lines, see for example the ‘*Decreto Rilancio*’ (Relaunch Decree), one of the major interventions by the government to tackle the economic consequences of the COVID-19 emergency, which among other interventions exempted citizens from the payment of local taxes such as the municipal property tax (*Imposta Municipale Unica*, IMU), the public land occupation tax (*Tassa* per *l'Occupazione del Suolo Pubblico*, TOSAP), and the regional tax on productive activities (*Imposta regionale sulle attività produttive*, IRAP). Details can be found here: https://www.mef.gov.it/en/inevidenza/Relaunch-Decree-155-billion-for-Phase-two-of-the-Economy-00001/.

6 Our sample does not include all 107 capitals of provinces due to constraints on the dependent variable, as discussed in the following section.

7 The poll was not conducted in all 107 capitals of provinces for different contingent reasons (e.g. the election for mayor was too close in time to the survey period). In our case, two municipalities were excluded in 2020 (Andria and Carbonia) and three in 2017 (Carbonia, Lodi, and Padua). To allow comparability between at least one data point during (2020) and before (2017) the COVID-19 pandemic, we limited the analysis to the subset of municipalities for which the poll is available in both periods. Therefore, our database covers 103 out of a total of 107 municipalities acting as capitals of provinces. We should also acknowledge that information for 2015 is limited to 98 of our 103 target municipalities.

8 The question was asked in Italian—the language of the respondents—as follows: ‘*Le chiedo un giudizio complessivo sull'operato del sindaco. Se domani ci fossero le elezioni comunali, lei voterebbe a favore o contro l'attuale sindaco?*‘.

9 The summary statistics for all variables used in the analysis are reported in Table A1 of the Online Appendix.

10 Specifically, the 2020 elections were held in October while the 2020 Governance Poll was conducted in June. In 2017, elections were held in June, while the 2017 Governance Poll was conducted in November–December 2016, and the 2015 elections were held in May, while the 2015 Governance Poll was conducted in March–April 2015.

11 The government also included *Italia Viva* (guided by Matteo Renzi, former Secretary of the Democratic Party) and *Liberi e Uguali* (a party from the left).

12 It is worth noting that these last variables (*education*, *unemployment rate,* and *firms*) are measured at the provincial level since information at the city level is only available from the 2011 census. Nevertheless, since our dataset includes only large cities, i.e. municipalities that are the capitals of a province (*capoluoghi di provincia*), we are confident that these measures represent a reasonable proxy for capturing socio-economic conditions at the city level.

13 In more detail, *gender* is a dummy variable that is equal to one if the mayor is female and zero otherwise. *Edu*, is a categorical variable that captures the level of education of the mayor, corresponding to 1 for having obtained a middle school diploma, 2 for a high school diploma, and 3 for a bachelor's degree and higher. *Profession* is a categorical variable ranging from 1 to 8, where 1 = teachers and educators; 2 = high-level professions (engineers, doctors, etc.); 3 = police and military; 4 = managers; 5 = employees; 6 = freelancers and entrepreneurs; 7 = unemployed, job seekers, and retirees; 7 others. Lastly, *vote margin* is the difference in terms of vote share obtained by the first (elected) and the second most voted (not elected) candidate.

14 We replicated our estimates by using the Governance Poll score indicators in percentages as the dependent variable. The findings, shown in Table A2 of the Online Appendix, yield very similar conclusions. Moreover, results based on errors clustered at the regional level are reported in Table A3 of the Online Appendix. Reassuringly, these are very close to those found with errors clustered at the municipality level.

15 The excess mortality indicator at the municipality level (source: ISTAT) is built as the difference between the average number of deaths in the first half of 2020 and the average number of deaths in the first half of the years 2015, 2016, 2017, 2018, and 2019. The number of COVID-19 cases indicator is constructed as the ratio between the monthly average of COVID-19 cases since the beginning of the pandemic and up to June 2020 at the provincial level (source: Italian Department of Civil Protection, ‘*Dipartimento della Protezione Civile’*; data retrieved from the following public repository: https://github.com/pcm-dpc/COVID-19/tree/master/dati-province) over the total population (source: ISTAT). Lastly, following Borri et al. (2021) the lockdown intensity indicator at the municipal level is proxied by the share of employees working for firms belonging to the economic activity (ATtività ECOnomica, ATECO) sectors that were not allowed to operate based on the Italian government decrees introducing the lockdown measures (in March 2020) over the total number of workers (source: ISTAT).

16 Following point estimates of Col. 1 – Table 4, the impact of being politically aligned during the pandemic for a city not highly exposed to the COVID-19 amounts to – 0.001–0.120 = – 0.121, which is statistically significant at 5%. The same effect for a city severely hit by the virus is equal to – 0.001–0.120 + 0.128 = 0.007, a not statistically significant estimation. This last finding, in practice, reveals that we do not observe any statistically significant difference in the Governance Poll indicators between aligned and not-aligned cities in areas severely hit by the COVID-19, before and during the pandemic.
