# Supplementary material for: Political alignment, centralisation, and the sense of government unpreparedness during the COVID-19 pandemic
Source: Eur J Polit Econ. 2022 Jun;73:None. doi: 10.1016/j.ejpoleco.2021.102144 (PMC8596202; doi:10.1016/j.ejpoleco.2021.102144)
Supplement: Multimedia component 1 [file mmc1.docx]

**Political alignment, centralisation, and the sense of government unpreparedness during the COVID-19 pandemic**

Supplementary Information

Supplementary Information Table of Contents

A. Descriptive Statistics 3

B. Robustness tests 6

C. Heterogeneous effects 12

**A Descriptive statistics**

**Table A1: Summary statistics**

|  |  |  |  |  |  |
| --- | --- | --- | --- | --- | --- |
| **Variables** | **Obs.** | **Mean** | **Std. Dev.** | **Min** | **Max** |
| (Log of) poll | 304 | 3.978 | 0.094 | 3.640 | 4.247 |
| Poll | 304 | 53.620 | 4.941 | 38.1 | 69.9 |
| Aligned | 304 | 0.599 | 0.491 | 0 | 1 |
| Post | 304 | 0.339 | 0.474 | 0 | 1 |
| Population | 304 | 171,021 | 332,183 | 21,457 | 2,873,494 |
| Children | 304 | 0.046 | 0.005 | 0.032 | 0.059 |
| Aged | 304 | 0.243 | 0.023 | 0.170 | 0.292 |
| Foreigners | 304 | 0.136 | 0.090 | 0.001 | 0.434 |
| Education | 304 | 0.129 | 0.076 | 0.028 | 0.481 |
| Unemployment rate | 304 | 11.179 | 5.794 | 3.014 | 31.456 |
| Firms | 304 | 39,570 | 18,975 | 7,699 | 114,035 |
| Age | 304 | 53.974 | 9.764 | 32 | 85 |
| Gender | 304 | 0.658 | 0.248 | 0 | 1 |
| Edu | 304 | 2.813 | 0.416 | 1 | 3 |
| Profession | 304 | 4.967 | 2.228 | 1 | 8 |
| Vote-margin | 304 | 18.506 | 15,045 | 0.351 | 95.137 |
| Excess of mortality | 103 | 17.855 | 53.511 | -85.267 | 452 |
| COVID-19 related cases (over pop) | 103 | 0.310 | 0.362 | 0.014 | 2.030 |
| High restrictions | 103 | 0.465 | 0.053 | 0.325 | 0.663 |

**Table A2: Baseline results using the dependent variable in percentage terms**

| Dep. Variable | Governance poll score | | | | | | | | |
| --- | --- | --- | --- | --- | --- | --- | --- | --- | --- |
|  | Panel A  Full Sample | | | |  | Panel B  Always vs. never aligned municipalities | | | |
|  | (1) | (2) | (3) | (4) |  | (5) | (6) | (7) | (8) |
|  |  |  |  |  |  |  |  |  |  |
| Aligned × Post | -3.406** | -3.199** | -2.625* | -3.265* |  | -4.773** | -5.034** | -3.974* | -5.862 |
|  | (1.502) | (1.501) | (1.388) | (1.925) |  | (1.934) | (2.127) | (2.105) | (4.479) |
| Aligned | 0.301 | 0.378 | -1.230 | -0.853 |  |  |  |  |  |
|  | (1.253) | (1.303) | (1.248) | (1.438) |  |  |  |  |  |
| Post | 0.953 | 1.606 | 0.391 | 1.531 |  | 1.560 | 3.271 | 0.017 | 0.173 |
|  | (1.085) | (2.563) | (2.562) | (4.093) |  | (1.480) | (3.380) | (3.572) | (6.045) |
| Observations | 304 | 304 | 304 | 304 |  | 134 | 134 | 134 | 134 |
| Adjusted R-squared | 0.030 | 0.054 | 0.239 | 0.259 |  | 0.092 | 0.115 | 0.302 | 0.350 |
| Municipality FE | Yes | Yes | Yes | Yes |  | Yes | Yes | Yes | Yes |
| Poll FE | Yes | Yes | Yes | Yes |  | Yes | Yes | Yes | Yes |
| Eco-socio-demographic controls | No | Yes | Yes | Yes |  | No | Yes | Yes | Yes |
| Political controls | No | No | Yes | Yes |  | No | No | Yes | Yes |
| Region × Poll | No | No | No | Yes |  | No | No | No | Yes |
|  |  |  |  |  |  |  |  |  |  |

**Note**: *Aligned* is a dummy variable that takes the value of one if the political party of the mayor belongs to the same political sphere as the national government and zero otherwise. *Post* is a dummy variable that takes on the value of one for the 2020 poll (during the pandemic) and zero otherwise. *Aligned x Post* is an interaction term equal to one for each municipality governed by a mayor politically aligned with the national government during the pandemic and zero otherwise. Eco-socio-demographic control variables are population, children, aged, foreigners, share of population enrolled at university, unemployment rate, and number of firms. Political control variables are mayor’s age, gender, education level, profession and distance in terms of vote share to the first non-elected candidate. Standard errors, clustered at the municipality level, are shown in parentheses. ***, **, and * indicate significance at the 1%, 5%, and 10% level, respectively.

**Table A3: Baseline results using errors clustered at the regional level**

| Dep. Variable | (Log of) governance poll score | | | |
| --- | --- | --- | --- | --- |
|  | (1) | (2) | (3) | (4) |
|  |  |  |  |  |
| Aligned × Post | -0.068* | -0.064* | -0.054* | -0.066 |
|  | (0,037) | (0.034) | (0.034) | (0.045) |
| Aligned | 0.007 | 0.009 | -0.020 | -0.013 |
|  | (0.030) | (0.030) | (0.030) | (0.039) |
| Post | 0.018 | 0.030 | 0.006 | 0.033 |
|  | (0.020) | (0.035) | (0.039) | (0.040) |
| Observations | 304 | 304 | 304 | 304 |
| Adjusted R-squared | 0.033 | 0.066 | 0.242 | 0.253 |
| Municipality FE | Yes | Yes | Yes | Yes |
| Poll FE | Yes | Yes | Yes | Yes |
| Eco-socio-demographic controls | No | Yes | Yes | Yes |
| Political controls | No | No | Yes | Yes |
| Region × Poll | No | No | No | Yes |
|  |  |  |  |  |

**Note**: *Aligned* is a dummy variable that takes the value one if the political party of the mayor belongs to the same political sphere as the national government and zero otherwise. *Post* is a dummy variable that takes the value of one for the 2020 poll (during the pandemic) and zero otherwise. *Aligned x Post* is an interaction term equal to one for each municipality governed by a mayor politically aligned with the national government during the pandemic and zero otherwise. *Eco-socio-demographic* control variables are population, children, aged, foreigners, share of population enrolled at university, unemployment rate, and number of firms. *Political* control variables are mayor’s age, gender, education level, profession and distance in terms of vote share to the first non-elected candidate. Standard errors, clustered at the regional level, are shown in parentheses. ***, **, and * indicate significance at the 1%, 5%, and 10% level, respectively.

**B Robustness tests**

Table B1: Results on the sample of always vs never aligned municipalities

| Dep. Variable | (Log of) governance poll score | | | |
| --- | --- | --- | --- | --- |
|  | (1) | (2) | (3) | (4) |
|  |  |  |  |  |
| Aligned × Post | -0.093** | -0.099** | -0.080* | -0.119 |
|  | (0.036) | (0.041) | (0.040) | (0.087) |
| Post | 0.029 | 0.063 | 0.001 | -0.004 |
|  | (0.027) | (0.065) | (0.070) | (0.118) |
| Observations | 134 | 134 | 134 | 134 |
| Adjusted R-squared | 0.095 | 0.129 | 0.306 | 0.344 |
| Municipality FE | Yes | Yes | Yes | Yes |
| Poll FE | Yes | Yes | Yes | Yes |
| Eco-socio-demographic controls | No | Yes | Yes | Yes |
| Political controls | No | No | Yes | Yes |
| Region × Poll | No | No | No | Yes |
|  |  |  |  |  |

**Note**: *Aligned* is a dummy variable that takes the value of one if the political party of the mayor belongs to the same political sphere as the national government and zero otherwise. *Post* is a dummy variable that takes on the value of one for the 2020 poll (during the pandemic) and zero otherwise. *Aligned x Post* is an interaction term equal to one for each municipality governed by a mayor politically aligned with the national government during the pandemic and zero otherwise. *Eco-socio-demographic* control variables are population, children, aged, foreigners, share of population enrolled at university, unemployment rate, and number of firms. *Political* control variables are mayor’s age, gender, education level, profession, and distance in terms of vote share to the first non-elected candidate. Standard errors, clustered at the municipality level, are shown in parentheses. ***, **, and * indicate significance at the 1%, 5%, and 10% level, respectively.

Table B2: Fake treatment results

| Dep. Variable | (Log of) governance poll score | | | | | | | | |
| --- | --- | --- | --- | --- | --- | --- | --- | --- | --- |
|  | Panel A  Full Sample | | | |  | Panel B  Always vs. never aligned municipalities | | | |
|  | (1) | (2) | (3) | (4) |  | (5) | (6) | (7) | (8) |
|  |  |  |  |  |  |  |  |  |  |
| Aligned × (Fake) Post | -0.017 | -0.004 | 0.004 | -0.017 |  | 0.001 | 0.002 | 0.023 | 0.014 |
|  | (0.022) | (0.028) | (0.022) | (0.021) |  | (0.030) | (0.041) | (0.032) | (0.030) |
| Aligned | -0.047 | -0.048 | -0.095*** | -0.099*** |  |  |  |  |  |
|  | (0.036) | (0.036) | (0.030) | (0.024) |  |  |  |  |  |
| (Fake) Post | -0.003 | -0.010 | -0.011 | 0.038 |  | -0.026 | 0.001 | -0.011 | -0.030 |
|  | (0.020) | (0.033) | (0.025) | (0.045) |  | (0.024) | (0.031) | (0.030) | (0.060) |
| Observations | 201 | 201 | 201 | 201 |  | 89 | 89 | 89 | 89 |
| Adjusted R-squared | 0.066 | 0.065 | 0.266 | 0.407 |  | 0.049 | 0.123 | 0.265 | 0.588 |
| Municipality FE | Yes | Yes | Yes | Yes |  | Yes | Yes | Yes | Yes |
| Poll FE | Yes | Yes | Yes | Yes |  | Yes | Yes | Yes | Yes |
| Eco-socio-demographic controls | No | Yes | Yes | Yes |  | No | Yes | Yes | Yes |
| Political controls | No | No | Yes | Yes |  | No | No | Yes | Yes |
| Region × Poll | No | No | No | Yes |  | No | No | No | Yes |

**Note**: *Aligned* is a dummy variable that takes the value of one if the political party of the mayor belongs to the same political sphere as the national government and zero otherwise. (*Fake)* *Post* is a dummy variable that takes the value of one for the 2017 poll and zero otherwise. *Aligned x (Fake) Post* is an interaction term equal to one for each municipality governed by a mayor politically aligned with the national government during 2017 and zero otherwise. *Eco-socio-demographic* control variables are population, children, aged, foreigners, share of population enrolled at university, unemployment rate, and number of firms. *Political* control variables are mayor’s age, gender, education level, profession, and distance in terms of vote share to the first non-elected candidate. Standard errors, clustered at the municipality level, are shown in parentheses. ***, **, and * indicate significance at the 1%, 5%, and 10% level, respectively.

Table B3: Further placebo test

| Dep. Variable | (Log of) governance poll score | | | | | | | | |
| --- | --- | --- | --- | --- | --- | --- | --- | --- | --- |
|  | Panel A  Full Sample | | | |  | Panel B  Always vs. never aligned municipalities | | | |
|  | (1) | (2) | (3) | (4) |  | (5) | (6) | (7) | (8) |
|  |  |  |  |  |  |  |  |  |  |
| Aligned × Year_2020_ | -0.057* | -0.058** | -0.050* | -0.059 |  | -0.092** | -0.098** | -0.080* | -0.127 |
|  | (0.029) | (0.029) | (0.028) | (0.040) |  | (0.039) | (0.043) | (0.043) | (0.091) |
| Aligned | -0.005 | 0.002 | -0.025 | -0.022 |  |  |  |  |  |
|  | (0.024) | (0.025) | (0.025) | (0.029) |  |  |  |  |  |
| Year_2020_ | 0.019 | 0.030 | 0.015 | 0.024 |  | 0.054* | 0.077 | 0.024 | 0.012 |
|  | (0.019) | (0.043) | (0.043) | (0.057) |  | (0.028) | (0.063) | (0.066) | (0.059) |
| Aligned × Year_2015_ | 0.029 | 0.017 | 0.010 | 0.019 |  | 0.001 | 0.002 | 0.001 | -0.024 |
|  | (0.022) | (0.026) | (0.022) | (0.025) |  | (0.030) | (0.037) | (0.027) | (0.035) |
| Year_2015_ | -0.012 | -0.005 | 0.006 | -0.012 |  | 0.025 | 0.014 | 0.023 | 0.019 |
|  | (0.019) | (0.022) | (0.019) | (0.036) |  | (0.023) | (0.026) | (0.021) | (0.035) |
| Observations | 304 | 304 | 304 | 304 |  | 134 | 134 | 134 | 134 |
| Adjusted R-squared | 0.034 | 0.065 | 0.240 | 0.252 |  | 0.088 | 0.122 | 0.300 | 0.339 |
| Municipality FE | Yes | Yes | Yes | Yes |  | Yes | Yes | Yes | Yes |
| Poll FE | Yes | Yes | Yes | Yes |  | Yes | Yes | Yes | Yes |
| Eco-socio-demographic controls | No | Yes | Yes | Yes |  | No | Yes | Yes | Yes |
| Political controls | No | No | Yes | Yes |  | No | No | Yes | Yes |
| Region × Poll | No | No | No | Yes |  | No | No | No | Yes |
|  |  |  |  |  |  |  |  |  |  |

**Note**: *Aligned* is a dummy variable that takes the value of one if the political party of the mayor belongs to the same political sphere as the national government and zero otherwise. *Year_2020_* is a dummy variable that takes on the value of one for the 2020 poll (during the pandemic) and zero otherwise. *Year_2015_* is a dummy variable that takes on the value of one for the 2015 poll and zero otherwise. *Eco-socio-demographic* control variables are population, children, aged, foreigners, share of population enrolled at university, unemployment rate, and number of firms. *Political* control variables are mayor’s age, gender, education level, profession and distance in terms of vote share to the first non-elected candidate. Standard errors, clustered at the municipality level, are shown in parentheses. ***, **, and * indicate significance at the 1%, 5%, and 10% level, respectively.

**Table B4: Control variables interacted with treatment (post)**

| Dep. Variable | (Log of) governance poll score | | Governance poll score | |
| --- | --- | --- | --- | --- |
|  | (1) | (2) | (3) | (4) |
|  |  |  |  |  |
| Aligned × Post | -0.043* | -0.043 | -2.155* | -2.318 |
|  | (0.026) | (0.039) | (1.275) | (2.108) |
| Aligned | -0.011 |  | -0.735 |  |
|  | (0.023) |  | (1.213) |  |
| Post | -0.257 | 0.745 | -15.761 | 37.413 |
|  | (0.484) | (0.936) | (25.280) | (49.881) |
| Population | 0.000*** | 0.000*** | 0.000*** | 0.000*** |
|  | (0.000) | (0.000) | (0.000) | (0.000) |
| Children | -6.300 | -2.776 | -322.705 | -173.975 |
|  | (7.285) | (13.063) | (379.362) | (689.241) |
| Aged | 1.342 | -4.220 | 66.971 | -221.611 |
|  | (2.343) | (5.197) | (122.492) | (263.835) |
| Foreigners | -0.019 | -0.201 | -1.132 | -10.727 |
|  | (0.191) | (0.344) | (10.186) | (18.547) |
| Education | 0.081 | 4.335** | 11.829 | 237.467** |
|  | (1.535) | (1.997) | (80.306) | (106.825) |
| Unemployment rate | 0.004 | 0.002 | 0.173 | 0.095 |
|  | (0.004) | (0.006) | (0.190) | (0.314) |
| Firms | 0.000 | 0.000** | 0.000 | 0.002** |
|  | (0.000) | (0.000) | (0.000) | (0.001) |
| Age | -0.001 | -0.002 | -0.074 | -0.140 |
|  | (0.001) | (0.002) | (0.068) | (0.121) |
| Gender | -0.055 | -0.061 | -2.706 | -3.993 |
|  | (0.034) | (0.102) | (1.726) | (5.568) |
| Edu | -0.014 | 0.047 | -0.686 | 2.507 |
|  | (0.026) | (0.037) | (1.377) | (2.010) |
| Profession | 0.009* | -0.002 | 0.491* | -0.120 |
|  | (0.005) | (0.009) | (0.267) | (0.499) |
| Vote-margin | 0.004*** | 0.003** | 0.199*** | 0.187*** |
|  | (0.001) | (0.001) | (0.040) | (0.066) |
| Population × Post | -0.000* | -0.000*** | -0.000 | -0.000*** |
|  | (0.000) | (0.000) | (0.000) | (0.000) |
| Children × Post | -3.034 | -17.3874 | -127.424 | -887.284 |
|  | (5.250) | (11.625) | (266.700) | (619.745) |
| Aged × Post | 1.049 | -0.832 | 59.433 | -40.871 |
|  | (1.043) | (1.957) | (54.806) | (103.970) |
| Foreigners × Post | 0.402 | 0.261 | 18.926 | 11.288 |
|  | (0.271) | (0.328) | (13.996) | (17.618) |
| Education × Post | -0.224 | -1.573 | -12.507 | -86.125 |
|  | (0.482) | (1.018) | (24.957) | (55.733) |
| Unemployment rate × Post | 0.004 | 0.009* | 0.209 | 0.493* |
|  | (0.004) | (0.005) | (0.202) | (0.256) |
| Firms × Post | 0.000 | 0.000 | 0.000 | 0.000 |
|  | (0.000) | (0.000) | (0.000) | (0.000) |
| Age × Post | 0.001 | 0.004 | 0.035 | 0.189 |
|  | (0.001) | (0.003) | (0.076) | (0.145) |
| Gender × Post | 0.011 | -0.041 | 0.337 | -1.768 |
|  | (0.043) | (0.082) | (2.336) | (4.490) |
| Edu × Post | 0.017 | 0.011 | 1.070 | 0.809 |
|  | (0.025) | (0.055) | (1.272) | (2.812) |
| Profession × Post | -0.011 | -0.003 | -0.593** | -0.213 |
|  | (0.005) | (0.011) | (0.263) | (0.583) |
| Vote-margin × Post | 0.001 | 0.003* | 0.022 | 0.132* |
|  | (0.001) | (0.001) | (0.032) | (0.077) |
| Observations | 304 | 134 | 304 | 134 |
| Adjusted R-squared | 0.283 | 0.431 | 0.275 | 0.415 |
| Municipality FE | Yes | Yes | Yes | Yes |
| Poll FE | Yes | Yes | Yes | Yes |
| Sample | Full | Always vs Never al. | Full | Always vs Never al. |
|  |  |  |  |  |

**Note**: *Aligned* is a dummy variable that takes the value of one if the political party of the mayor belongs to the same political sphere as the national government and zero otherwise. *Post* is a dummy variable that takes on the value of one for the 2020 poll (during the pandemic) and zero otherwise. *Aligned x Post* is an interaction term equal to one for each municipality governed by a mayor politically aligned with the national government during the pandemic and zero otherwise. Standard errors, clustered at the municipality level, are shown in parentheses. ***, **, and * indicate significance at the 1%, 5%, and 10% level, respectively.

**Table B5: Balancing test**

| Dep. Variable | Population | Children | Aged | Foreigners | Education | Unemployment rate | Firms | Age | Gender | Edu | Profession | Vote-margin |
| --- | --- | --- | --- | --- | --- | --- | --- | --- | --- | --- | --- | --- |
|  | (1) | (2) | (3) | (4) | (5) | (6) | (7) | (8) | (9) | (10) | (11) | (12) |
|  |  |  |  |  |  |  |  |  |  |  |  |  |
| Aligned × Post | -335.316 | -0.000 | -0.001 | -0.002 | 0.001 | -0.683 | -17.222 | 1.636 | 0.048 | 0.045 | 0.317 | -2.192 |
|  | (1577.628) | (0.000) | (0.001) | (0.011) | (0.001) | (0.537) | (230.874) | (2.114) | (0.045) | (0.106) | (0.492) | (3.482) |
| Aligned | -616.104 | -0.000* | -0.001 | 0.001 | 0.000 | 0.163 | 452.726* | -1.921 | -0.049) | 0.051 | -0.580 | 8.441*** |
|  | (720.257) | (0.000) | (0.001) | (0.007) | (0.001) | (0.372) | (266.128) | (2.278) | (0.055) | (0.079) | (0.466) | (2.839) |
| Post | 1133.988 | -0.003*** | 0.007*** | 0.133*** | -0.002 | -1.908*** | -63.972 | 1.936 | -0.067 | -0.006 | 0.712 | 3.754 |
|  | (2291.842) | (0.000) | (0.002) | (0.013) | (0.003) | (0.666) | (396.793) | (3.129) | (0.117) | (0.126) | (0.907) | 5.802 |
|  |  |  |  |  |  |  |  |  |  |  |  |  |
| Observations | 304 | 304 | 304 | 304 | 304 | 304 | 304 | 304 | 304 | 304 | 304 | 304 |
| Adjusted R-squared | 0.072 | 0.868 | 0.787 | 0.782 | 0.760 | 0.515 | 0.371 | 0.104 | 0.042 | 0.025 | 0.085 | 0.174 |
| Municipality FE | Yes | Yes | Yes | Yes | Yes | Yes | Yes | Yes | Yes | Yes | Yes | Yes |
| Poll FE | Yes | Yes | Yes | Yes | Yes | Yes | Yes | Yes | Yes | Yes | Yes | Yes |
| Controls | Yes | Yes | Yes | Yes | Yes | Yes | Yes | Yes | Yes | Yes | Yes | Yes |

**Note**: *Aligned* is a dummy variable that takes the value of one if the political party of the mayor belongs to the same political sphere as the national government, and zero otherwise. *Post* is a dummy variable that takes the value of one for the 2020 poll (during the pandemic) and zero otherwise. *Aligned x Post* is an interaction term equal to one for each municipality governed by a mayor politically aligned with the national government during the pandemic and zero otherwise. Control variables are population, children, aged, foreigners, share of population enrolled at university, unemployment rate, number of firms, mayor’s age, gender, education level, profession, and distance in terms of vote share to the first non-elected candidate, and exclude the dependent variable. Standard errors, clustered at the municipality level, are shown in parentheses. ***, **, and * indicate significance at the 1%, 5%, and 10% level, respectively.

Figure B1: Municipality excluded

.

**C Heterogeneous effects**

To investigate whether there is evidence of a heterogeneous response to the shift in the policy-making decision system, we analyse how the effect varies along several dimensions.

*Pre-electoral years*

The political economy literature (Rogoff, 1990; Rogoff and Sibert, 1988) has shown that policymakers have an incentive to manipulate policy decisions close to elections, and such an incentive is found to be particularly strong at the local level (Akhmedov and Zhuravskaya, 2004; Drazen and Eslava, 2010; Kneebon and McKenzie, 2001). As for the specific case of Italian municipalities, the empirical evidence has documented the existence of political budget cycles, considering both taxes (Alesina and Paradisi, 2017) and expenditure (see Ferraresi et al. (2019) for the case of current expenditure, and Repetto (2018) for the case of investment expenditure). Therefore, in standard times, when municipalities enjoy the usual discretion in setting policies, it is very likely that such a strategic incentive to manipulate policy decisions is higher when elections get close. The governance score would then be influenced by this strategic behaviour. In other words, when cities are close to holding elections, policy makers have an incentive to manipulate fiscal policy by focusing their efforts on visible actions that could be reflected in a higher governance score.

But what happens when decisions are centralised? Does this incentive still hold? To test whether the alignment status is affected by political-cycle incentives once the decision-making process is centralised, we build a dummy variable, *Pre-Election*, equal to one in the year before an election and zero otherwise. We then interact it with both *Aligned* and *Pre-Aligned × Post* in a triple-difference model. Hence, the model we estimate, which is a generalized version of that in Eq. (1), takes the following form:

$${Governancepollscore}_{it}=\alpha+\beta{Aligned}_{it}+\lambda{Post}_{t}+\gamma{Aligned}_{it}\times{Post}_{t}+\delta{Aligned}_{it}\times{{Pre}_{Election}}_{it}+\theta{Post}_{it}\times{{Pre}_{Election}}_{it}+\rho{{PR}_{Election}}_{it}+\eta{Aligned}_{it}\times{Post}_{t}\times{{Pre}_{Election}}_{it}+f_{t}+f_{i}+u_{it}.$$

Our variables of interest are ${Aligned}_{it}\times{Post}_{t}$ and ${Aligned}_{it}\times{Post}_{t}\times{{Pre}_{Election}}_{it}$. The former term captures the differential impact of being under a centralized system when outside of pre-electoral years for aligned with respect to not aligned municipalities minus the difference of being not in a centralized system when outside of pre-electoral years for aligned with respect to not aligned municipalities.

Shown in column 1 of Table C1, the coefficient of ${Aligned}_{it}\times{Post}_{t}$ is negative but not statistically significant. Turning now to the triple-interaction term, ${Aligned}_{it}\times{Post}_{t}\times{{Pre}_{Election}}_{it}$, it is found to be negative (–0.132) and statistically significant at the 10% level. What these results simply reveal is that aligned municipalities that were in a pre-electoral year during the lockdown experienced a larger decrease in the governance indicator as compared to municipalities that would not run for elections in the following year.

*Strong mandates*

Local government councils backed by large majorities are expected to be relatively confident of re-election as they enjoy much visibility and, therefore, it might be unlikely that citizens are unable to discriminate the extent to which the policies adopted by the mayor are the result of his/her own mandate. In other words, if the mayor is very strong in terms of political consensus, local policy outcomes—and thus the governance indicator—are expected to be well distinguished from the central ones. In contrast, such a distinction might not be so clear-cut for mayors backed by a narrow majority.

In order to test this hypothesis, we gather information on the share of votes that were necessary to win an election, in order to measure the political power of the mayor. In particular, we use the median share of votes (approximately 60%) to create a dummy variable, *Large Consensus*, that divides municipalities into those in which the mayor is supported by a small majority (below the sample median) and those in which the mayor is supported by a large majority (above the sample median). We then interact it with both *Aligned* and *Aligned × Post* in a triple-difference model that takes the following form:

$${Governancepollscore}_{it}=\alpha+\beta{Aligned}_{it}+\lambda{Post}_{t}+\gamma{Aligned}_{it}\times{Post}_{t}+\delta{Aligned}_{it}\times{LargeConsensus}_{it}+\theta{Post}_{it}\times{LargeConsensus}_{it}+\rho{LargeConsensus}_{it}+\eta{Aligned}_{it}\times{Post}_{t}\times{LargeConsensus}_{it}+f_{t}+f_{i}+u_{it}.$$

As before, our coefficients of interest are *γ* and $\eta$. The first captures the differential impact for aligned municipalities and for mayors not supported by larger majorities, of being under a centralised decision system. Instead, the second captures the differential effect for mayors supported by a large consensus.

Our estimates presented in column (2) of Table C1 document that the coefficient of *Aligned × Post* is negative (–0.061) and statistically significant at the 5% level, while that of *Aligned × Post × Large Consensus* is not statistically significant. This suggests that the governance score of aligned municipalities decreases when the policy decision process is entirely in the hands of the central government, regardless of the status—small vs large majority—of their mayor.

*Social capital*

The concept of social capital is often associated with the presence of a certain degree of interpersonal (Fukuyama, 1995) or generalized trust (Putnam, 2000) within a society. Nannicini et al. (2013) also provide a definition related to participation in political life: the larger is the share of voters who care more about aggregate rather than individual welfare, the higher can be considered their social capital. Based on this definition, they find that voters with lower social capital tend to punish their representatives less for their poor performance. In our setting, we want to further test whether different levels of social capital lead to heterogenous opinions about the local authorities in the cases in which the mayor is politically aligned, or not, with the national government.

Like Nannicini et al. (2013), we adopt the per capita level of blood donations as a proxy for social capital, for two main reasons: On the one hand, in Italy donating blood is a substantially altruistic deed since no monetary incentives are in place for donors. On the other hand, given that the donation is anonymous, even non-monetary payoffs linked to social common approval should be limited. We then create a dummy variable, *High Social Capital*, that divides municipalities into those in which the social capital is high (above the sample median) and those in which it is low (below the sample median), based on provincial levels in 2001.^[[1]](#footnote-1)^ As in previous estimations, we then interact it with both *Aligned* and *Pre-Aligned × Post* in a triple-difference model as follows:

$${Governancepollscore}_{it}=\alpha+\beta{Aligned}_{it}+\lambda{Post}_{t}+\gamma{Aligned}_{it}\times{Post}_{t}+\delta{Aligned}_{it}\times{HighSocialCapital}_{i}+\theta{Post}_{it}\times{HighSocialCapital}_{i}+\eta{Aligned}_{it}\times{Post}_{t}\times{HighSocialCapital}_{i}+f_{t}+f_{i}+u_{it}.$$

We are mostly interested in the coefficient *γ*, capturing the differential impact for aligned municipalities with lower levels of social capital, and $\eta$, capturing the differential effect for municipalities with higher levels of social capital.

We find that voters in municipalities with a lower level of social capital are more likely to punish local politicians who are politically aligned with the national government. Indeed, our estimates in column (3) of Table C1 show that the coefficient of *Aligned × Post* is negative (–0.097) and significant, while the coefficient of *Aligned × Post × High Social Capital* is positive (+0.098) and significant. At the same time, the overall effect on municipalities with higher levels of social capital amounts to (0.004 – 0.097 + 0.098) = 0.005, which is not statistically different from zero (p-value = 0.278). In other words, the reduction in governance score for aligned municipalities is different based on the level of social capital but is only significant (and negative) in case of lower levels of social capital, while there is no effect for higher levels.

These results document that voters who are more interested in collective well-being better discriminate the political activity of the different government layers, while more individualistic voters tend to confuse political responsibilities across governmental levels.

*Central vs Regional government alignment*

To provide additional evidence in favour of the *punishment channel* hypothesis, we collect information on the party affiliation of the regional council over the period of 2015–2020 and we create an additional dummy variable that is equal to 1 if the mayor's coalition is the same as the coalition in power at the regional level, and zero otherwise (*Aligned_Reg_*). These variables are then interacted with *Post* in the following triple difference model:

${Governancepollscore}_{it}=\alpha+\beta{Aligned}_{it}+\lambda{Post}_{t}+\gamma{Aligned}_{it}\times{Post}_{t}+{\pi AlignedReg}_{it}+\rho{Post}_{it}\times{AlignedReg}_{it}+{\delta Aligned}_{it}\times{Post}_{t}\times{AlignedReg}_{it}{+f}_{t}+f_{i}+u_{it}.$

While the government decision-making process became highly centralised during the pandemic, regions were entitled to set additional (and more restrictive) measures.^[[2]](#footnote-2)^ Therefore, the split of the aligned coefficient would allow us to understand which of the two effects prevails. On the one hand, if the centralisation of policy-making decisions triggered by the pandemic brought about citizen confusion regarding policy responsibilities across different levels of government, we would expect both the coefficients *γ* and $\delta$ to be negative and statistically significant. On the other hand, if the decline of the governance indicator observed in aligned cities captures negative perceptions of the policies adopted by the central government, including measures that restrict the movement of individuals (e.g. the ‘lockdown’), we would observe a negative and significant coefficient associated with municipalities aligned only with the government, and no effect for cities aligned with the regional council ($\delta$). In practice, following this specification, the impact on the governance poll score for cities aligned with the central government (but not with the regional one) during the pandemic is given by $\beta+\gamma$, whereas the same effect for cities also aligned with the regional government is given by $\beta+\gamma+\delta$. Results of this analysis are reported in col. (4) and indicate that the drop in the governance pool indicator for cities that are also aligned with the regional government is less marked that that observed for cities aligned with the central government, albeit the $\delta$ coefficient is not statistically significant. Following point estimates in col. (4), it turns out that the impact on the governance indicator for aligned cities is equal to -0.127 (-0.127 = -0.065 – 0.062), an estimate statistically significant at 1%. The same impact for cities aligned also with the regional government yields an estimate equal to -0.111 (-0.111 = -0.065 – 0.062 + 0.016), which is statistically different from zero at 5%. Taken together, these results, if purely suggestive, reinforce the idea of the ‘punishment’ channel, as the decline of the governance indicator is more pronounced for municipalities aligned only with the government than for cities aligned with the regional council.

Table C1: Heterogeneous effects

| Dep. Variable | (Log of) governance poll score | | | |
| --- | --- | --- | --- | --- |
|  | (1) | (2) | (3) | (4) |
|  |  |  |  |  |
| Aligned | -0.020 | -0.004 | 0.004 | -0.065 |
|  | (0.023) | (0.032) | (0.032) | (0.036) |
| Post | 0.003 | -0.005 | 0.018 | 0.020 |
|  | (0.052) | (0.048) | (0.049) | (0.051) |
| Aligned × Post | -0.030 | -0.061** | -0.097** | -0.062 |
|  | (0.028) | (0.030) | (0.037) | (0.040) |
| Pre-election | -0.104** |  |  |  |
|  | (0.051) |  |  |  |
| Aligned × Pre-Election | 0.054 |  |  |  |
|  | (0.055) |  |  |  |
| Post × Pre-Election | 0.092 |  |  |  |
|  | (0.058) |  |  |  |
| Aligned × Post × Pre-Election | -0.132* |  |  |  |
|  | (0.074) |  |  |  |
| Large Consensus |  | 0.089*** |  |  |
|  |  | (0.033) |  |  |
| Aligned × Large Consensus |  | -0.054 |  |  |
|  |  | (0.041) |  |  |
| Post × Large Consensus |  | 0.014 |  |  |
|  |  | (0.033) |  |  |
| Aligned × Post × Large Consensus |  | 0.007 |  |  |
|  |  | (0.048) |  |  |
| Aligned × High Social Capital |  |  | -0.057 |  |
|  |  |  | (0.043) |  |
| Post × High Social Capital |  |  | -0.033 |  |
|  |  |  | (0.039) |  |
| Aligned × Post × High Social Capital |  |  | 0.098** |  |
|  |  |  | (0.048) |  |
| Regional Aligned |  |  |  | -0.039 |
|  |  |  |  | (0.045) |
| Aligned × Regional Aligned |  |  |  | 0.087 |
|  |  |  |  | (0.055) |
| Post × Regional Aligned |  |  |  | 0.002 |
|  |  |  |  | (0.047) |
| Aligned × Post × Regional Aligned |  |  |  | 0.016 |
|  |  |  |  | (0.059) |
| Observations | 304 | 304 | 304 | 304 |
| Adjusted R-squared | 0.298 | 0.331 | 0.254 | 0.261 |
| Municipality FE | Yes | Yes | Yes | Yes |
| Poll FE | Yes | Yes | Yes | Yes |
| Eco-socio-demographic controls | Yes | Yes | Yes | Yes |
| Political controls | Yes | Yes | Yes | Yes |

**Note**: *Aligned* is a dummy variable that takes the value of one if the political party of the mayor belongs to the same political sphere as the national government and zero otherwise. *Post* is a dummy variable that takes the value of one for the 2020 poll (during the pandemic) and zero otherwise. *Aligned x Post* is an interaction term equal to one for each municipality governed by a mayor politically aligned with the national government during the pandemic and zero otherwise. *Pre-Election* is a dummy variable that takes the value of one in the year before the municipality election and zero otherwise. *Aligned × Pre-Election* is an interaction term equal to one if the political party of the mayor belongs to the same political sphere as the national government for municipalities in the year before the election and zero otherwise. *Post × Pre-Election* is an interaction term equal to one for municipalities in the year before the election during the pandemic and zero otherwise. *Aligned × Post × Pre-Election* is an interaction term equal to one for municipalities governed by a mayor politically aligned with the national government during the pandemic in a pre-electoral year and zero otherwise. *Large Consensus* is a dummy variable that takes the value of one for municipalities in which the mayor was elected with more than 50% of votes in the previous elections and zero otherwise. *Aligned × Large Consensus* is an interaction term equal to one for municipalities in which the mayor was elected with more than 50% of votes in the previous election and is aligned with the national government, and zero otherwise. *Post × Large Consensus* is an interaction term equal to one for municipalities in which the mayor was elected with more than 50% of votes in the previous election during the pandemic and zero otherwise. *Aligned × Post × Large Consensus* is an interaction term equal to one for municipalities governed by a mayor elected with more than 50% of votes in the previous election and politically aligned with the national government during the pandemic, and zero otherwise. *High Social Capital* is a dummy variable that takes the value of one for municipalities with a level of social capital larger than the median value and zero otherwise. *Aligned × High Social Capital* is an interaction term equal to one if the political party of the mayor belongs to the same political sphere as the national government for municipalities with a high level of social capital and zero otherwise. *Post × High Social Capital* is an interaction term equal to one for municipalities with a high level of social capital during the pandemic and zero otherwise. *Aligned × Post × High Social Capital* is an interaction term equal to one for municipalities with a high level of social capital governed by a mayor politically aligned with the national government during the pandemic, and zero otherwise. *Regional Aligned* is a dummy variable that takes the value of one if the political party of the mayor belongs to the same political sphere as the regional government and zero otherwise. *Aligned × Regional Aligned* is an interaction term equal to one if the political party of the mayor belongs to the same political sphere as the regional and the national governments, and zero otherwise. *Post × Regional Aligned* is an interaction term equal to one for municipalities with a mayor belonging to the same political sphere as the regional government during the pandemic, and zero otherwise. *Aligned × Post × Regional Aligned i*s an interaction term equal to one for municipalities with a mayor belonging to the same political sphere of the regional and national governments during the pandemic, and zero otherwise. *Eco-socio-demographic* control variables are population, children, aged, foreigners, share of population enrolled at university, unemployment rate, and number of firms. *Political* control variables are mayor’s age, gender, education level, profession, and distance in terms of vote share to the first non-elected candidate. Standard errors, clustered at the municipality level, are shown in parentheses. ***, **, and * indicate significance at the 1%, 5%, and 10% level, respectively.

**References**

Akhmedov, A., & Zhuravskaya, E. (2004). Opportunistic political cycles: test in a young democracy setting. *The Quarterly Journal of Economics*, *119*(4), 1301–1338.

Alesina, A., & Paradisi, M. (2017). Political budget cycles: Evidence from Italian cities. *Economics & Politics*, *29*(2), 157–177.

Drazen, A., & Eslava, M. (2010). Electoral manipulation via voter-friendly spending: Theory and evidence. *Journal of Development Economics*, *92*(1), 39–52.

Ferraresi, M., Galmarini, U., Rizzo, L., & Zanardi, A. (2019). Switch toward tax centralization in Italy: a wake-up for the local political budget cycle. *International Tax and Public Finance*, *26*(4), 872–898.

Fukuyama, F. (1995). Social capital and the global economy. *Foreign Aff.*, *74*, 89.

Kneebone, R.D., & McKenzie, K.J. (2001). Electoral and partisan cycles in fiscal policy: An examination of Canadian provinces. *International Tax and Public Finance*, *8*(5–6), 753–774.

Nannicini, T., Stella, A., Tabellini, G., & Troiano, U. (2013). Social capital and political accountability. *American Economic Journal: Economic Policy*, *5*(2), 222-50.

Putnam, R. D. (2000). *Bowling alone: The collapse and revival of American community*. Simon and schuster.

Repetto, L. (2018). Political budget cycles with informed voters: evidence from Italy. *The Economic Journal*, *128*(616), 3320–3353.

Rogoff, K. (1987). *Equilibrium political budget cycles* (No. w2428). National Bureau of Economic Research.

Rogoff, K., & Sibert, A. (1988). Elections and macroeconomic policy cycles. *The Review of Economic Studies*, *55*(1), 1–16.

1. A recent work by Daniele et al. (2020) has shown that the COVID-19 pandemic has brought declines in interpersonal and institutional trust, i.e. two proxies of social capital. For this reason, to avoid endogeneity issues, we measure social capital before the pandemic. [↑](#footnote-ref-1)
2. For example, at the beginning of the pandemic, while in contrast with the Decree of the Prime Minister, the President of Marche ordered the suspension of all public events and the closure of schools of all levels. More details on other restrictive measures adopted by regions can be found in the following link: [https://www.iusinitinere.it/il-controverso-rapporto-stato-regioni-nella-gestione-dellemergenza-sanitaria-27080#sdfootnote4sym](https://www.iusinitinere.it/il-controverso-rapporto-stato-regioni-nella-gestione-dellemergenza-sanitaria-27080#sdfootnote4sym). [↑](#footnote-ref-2)
